# Engineering student experience and self-direction in implementations of blended learning: a cross-institutional analysis

**DOI:** 10.1186/s40594-023-00406-x

**Published:** 2023-03-08

**Authors:** David Evenhouse, Yonghee Lee, Edward Berger, Jeffrey F. Rhoads, Jennifer DeBoer

**Affiliations:** 1 Department of Engineering and Computing Education, University of Cincinnati, Cincinnati, OH USA; 2 School of Engineering Education, Purdue University West Lafayette, West Lafayette, IN USA; 3 Mechanical Engineering Education Research Center at Purdue University (MEERCat Purdue), West Lafayette, IN USA; 4 School of Mechanical Engineering, Purdue University West Lafayette, West Lafayette, IN USA

**Keywords:** Blended learning, Instructional change, Student experience, Self-directed learning, Study behaviors

## Abstract

**Background:**

Much of researchers’ efforts to foster wider implementation of educational innovations in STEM has focused on understanding and facilitating the implementation efforts of faculty. However, student engagement in blended learning and other innovations relies heavily on students’ self-directed learning behaviors, implying that students are likely key actors in the implementation process. This paper explores the ways in which engineering students at multiple institutions experience the self-directed selection and implementation of blended learning resources in the context of their own studies. To accomplish this, it adopts a research perspective informed by Actor-Network Theory, allowing students themselves to be perceived as individual actors and implementors rather than a population that is implemented upon.

**Results:**

A thematic analysis was conducted in two parts. First, analysis identified sets of themes unique to the student experience at four participant institutions. Then, a second round of analysis identified and explored a subset of key actors represented in students’ reported experiences across all institutions. The findings show clear similarities and differences in students’ experiences of blended learning across the four institutions, with many themes echoing or building upon the results of prior research. Distinct institutional traits, the actions of the instructors, the components of the blended learning environment, and the unique needs and preferences of the students themselves all helped to shape students’ self-directed learning experiences. Students’ engagement decisions and subsequent implementations of blended learning resulted in personally appropriate, perhaps even idiosyncratic, forms of engagement with their innovative learning opportunities.

**Conclusion:**

The institutional implementation of blended learning, and perhaps other educational innovations, relies in part on the self-directed decision-making of individual students. This suggests that instructors too hold an additional responsibility: to act as facilitators of their students’ implementation processes and as catalysts for growth and change in students’ learning behaviors. Developing a greater understanding of students’ implementation behaviors could inform the future implementation efforts of faculty and better empower students to succeed in the innovative classroom.

## Introduction

Researchers in STEM education, and in the context of engineering more specifically, have long called for broader dissemination and application of research-based innovations in undergraduate teaching (Besterfield-Sacre et al., 2014; Borrego et al., 2013; Jamieson & Lohmann, 2012). This push for increased translation of research into practice has coincided with an increasing academic interest in the *implementation* of such innovations (Henderson et al., 2011; Reinholz et al., 2020). The resulting literature has been predominately focused on the actions and experiences of faculty, and for good reason: faculty act to enhance the learning opportunities available to their students through the implementation of educational innovations, and facilitating or streamlining that implementation process is integral to the dissemination of new innovations (Finelli & Borrego, 2020; Liu et al., 2020; Mirriahi et al., 2015). As a result, researchers have come to identify a variety of factors that influence the implementation of innovations among STEM instructors.

However, sole focus on this faculty-oriented perspective may limit our understanding of students’ experiences and the roles they play in the implementation process (Kezar et al., 2015). For the past six years, our research team has been collecting data on the implementation of a blended learning environment called *Freeform*. The *Freeform* environment was developed to combine a variety of research-based pedagogical innovations and learning resources (Rhoads et al., 2014) and has been implemented at several engineering institutions—both domestically within the USA and internationally—since its formal introduction in 2014 (Kandakatla et al., 2018). One of the major affordances of blended learning is flexibility, allowing students to determine how best to tailor resource use to fit their individual needs (Means et al., 2009). This flexibility, however, depends on student’s own self-directed engagement. Furthermore, in our prior work, we have seen student decision-making play a vital role in determining how the overall implementation of blended learning manifested in the behaviors, experiences, and outcomes for students and faculty alike (Evenhouse et al., 2018, 2020; Kandakatla et al., 2020; Stites et al., 2019).

A better understanding of how students engage with the innovative resources contained in blended learning environments could provide valuable insight for future implementations of hybrid and blended learning, which have become even more prevalent in the wake of COVID-19. In addition, we suspect that a deeper understanding of the student experience and recognition of students as central actors in implementation could inspire a change in perspective. As *Freeform* has been disseminated to institutions beyond our own, it has also been exposed to students of many different contexts and backgrounds, allowing us to examine the variation in student implementations of blended learning innovations across institutional types and cultures. The purpose of this paper is to further examine the role that students play in the practical implementation of their blended learning environment: not just as a factor influencing the actions of faculty, but as actors working with the innovation itself.

### Blended learning context: the learning environment

*Freeform* is a pedagogical system which has been iteratively developed, researched, and propagated through the joint effort of faculty in the Purdue University—West Lafayette (PUWL) Schools of Engineering Education (ENE) and Mechanical Engineering (ME). Originally conceived to enhance teaching and learning in 2nd year dynamics courses (Rhoads et al, 2014), the *Freeform* environment has since been applied to other courses in ME mechanics (Kandakatla et al., 2018) and other core engineering sciences (e.g., in Chemical Engineering). The environment was designed to combine best practices in blended learning (Halverson et al., 2014; Means et al., 2009) with active (Christie & de Graaff, 2017; Freeman et al., 2014) and collaborative (Barkley et al., 2014; Dillenbourg, 1999) instructional approaches, encouraging the adoption of innovative pedagogical methods and allowing students to engage with a wide variety of in-person and online learning resources (Rhoads et al., 2014). This is also the primary difference between the pedagogical environment and similar applications of educational technology, such as Learning Management Software (LMS) or enhanced textbooks—the physical and digital resources in the environment are intended to complement and facilitate the simultaneous use of various research-based teaching practices. The online resources also act as a supplement to in-class learning (Francis & Shannon, 2013), thereby promoting opportunities for self-regulated learning behavior outside of class (Zimmerman, 2001). Table 1 gives an overview of the learning resources typically available to students in *Freeform* courses on the PUWL campus.

**Table 1:** Learning resources in the *Freeform* environment (Evenhouse et al., 2020)

Resource	Contribution to the *Freeform* environment
Lecturebook	The lecturebook acts as the *Freeform* equivalent of a textbook. It contains ample white space so that students may take notes or write-out solutions alongside the text itself. The text includes equations, derivations, example problems, and conceptual questions
Instructor Office Hours	The instructor’s office hours are a predetermined set of times each week during which students ask questions about the course, its content, or their assessments
TA Help Room*	The Teaching Assistant (TA) help room, similar to instructor office hours, allows students to request help from course TAs at a predetermined location during a set schedule each week
Online Solution Videos**	*Freeform* maintains an online library of videos which provide step-by-step solutions to lecture example problems (contained in the lecturebook) and completed homework problems
Demonstration Videos**	Students were employed to create live-action videos for the *Freeform* environment which demonstrate real-world applications and embodiments of fundamental dynamics concepts. The videos are maintained online under the name Visualizing Mechanics
Discussion Forums**	Each class in the learning environment has a designated forum for online discussion and collaboration pertaining to homework assignments and exams
Peer Collaboration	Students were encouraged to collaborate with their peers from dynamics both inside and outside the classroom. Although this resource is not provided to students directly, it is facilitated throughout the course and remains an essential part of the *Freeform* environment

*The TA Help Room is a dedicated space on PUWL’s campus where students can find tutorial help during its daily operating hours. Other institutions employing the *Freeform* environment may not have TA Help Rooms of their own

**The Online Solution Videos, Demonstration Videos, and Discussion Forums are all made available through the Freeform course blog, along with homework assignments and general course information

Having these resources distributed to multiple implementing institutions provides a unique opportunity to study how and why students interact with specific subsets of resources and learning opportunities (Kandakatla et al., 2018). Previous work by Stites et al. (2019), Stites et al. (2020) used cluster analysis to identify nine distinct resource usage patterns among students. These patterns, with few exceptions, did not predict significant differences in academic performance. Rather, students chose to engage with resources they expected to best address their needs and could readily explain engagement decisions when interviewed (Evenhouse et al., 2020; Stites et al., 2019). The analyses and findings resulting from these students’ explanations echoed the conclusions of previous theories such as the Technology Acceptance Model (Davis, 1993) or the expectancy-value model (Makara & Karabenick, 2013). However, the manifest depth and variety of student experience further complicated our understanding of academic implementation. Students’ decisions were influenced by a host of factors comprising future goals, scheduling limitations, personal preferences, background factors, and prior educational experiences (Evenhouse et al., 2018, 2020; Kandakatla et al., 2020). Even personal context such as students’ housing situations could influence how they engaged with learning resources in their blended learning environment (Evenhouse et al., 2018, 2020).

Although we have evaluated the implementation of *Freeform* from a number of perspectives (Evenhouse et al., 2018; Kandakatla et al., 2018), we have been increasingly drawn to the experiences, behaviors, and demonstrated agency of students themselves. For example, in their discussion of resource usage and agency, Stites et al. (2019) encouraged faculty to consider allowing students to make their own implementation decisions, rather than expecting all students to use specific, tailored approaches to learning in the innovative classroom. Evenhouse et al. (2020) came to a similar conclusion, emphasizing that instructors should encourage mindsets and behaviors that promote deep learning, rather than attempting to prescribe specific methods and resources for all of their students to use. In implementing blended learning environments across diverse contexts, we expect students to play a pivotal role in the introduction, use, and long-term success of their learning innovations. To better understand the experiences and roles of students in the implementation of such innovations, we address the following research questions:

- RQ1: How do students experience and engage in implementation within the *Freeform* environment?
- RQ2: How do these experiences compare across institutionally distinct contexts?

## Literature review

### Implementation in undergraduate STEM

Borrego and Henderson (2014) introduced *implementation* in higher education as an intentional and targeted process of curricular change. Through implementation, faculty adopt, adapt, and subsequently integrate specific educational innovations to new environments via strategies designed to promote the innovations' efficacy. In this way, the decision to adopt a new research-based innovation is only the first step of any implementation (Taylor et al., 2018b; Tornatzky & Klein, 1982): faculty then apply, test, and iteratively adapt the innovation to fit with the unique needs and values of their classroom's context (Aarons, et al., 2019; Baumann et al., 2017). Ideally, these intentional processes of targeted change result in the successful, effective, and long-term use of research-based innovations (Moullin et al., 2019; Rogers, 2003), applied and adapted in ways that benefit both faculty and their students.

Henderson and Dancy (2007), Lattuca et al. (2009), and others model change in higher education using a combination of individual and environmental—or institutional—factors, each of which influence individual decision-making and shape the final form of a given implementation. In short, the process of implementation in education is shaped by its implementors and context (Lattuca et al., 2009). In this body of work, students are most often included as an environmental factor, as these models of implementation typically assume that the implementors in question are faculty or administrators. For example, in the 2014 *Journal of Engineering Education* special issue on systematic change in STEM higher education, students were almost exclusively represented in the form of *student resistance* as experienced by faculty members (McKenna et al., 2014).

It is the influence of these individual and environmental factors in implementation that often result in the development of local adaptations. Individual factors may require that implementors better adapt the innovation to fit with their personal needs, abilities, or preferences. For example, faculty may have strong conceptions of learning theory or a long history of educational experience, leading them to alter the ways in which they utilize innovations to better match their prior experience and skillset (Englund et al., 2017). In contrast, environmental factors could require implementors to adapt innovations to fit with the needs, values, or capabilities of their context. For example, faculty may have to adapt their use of new educational technologies to fit with their institutional infrastructure or their students’ preferences (Cohen & Ball, 2007; Henderson & Dancy, 2008). Such adaptations are a necessary part of the implementation process, allowing innovations to be applied broadly despite differences in institutional, cultural, or physical context (Dancy & Henderson, 2008). Thus, any adaptations to an educational innovation that are determined to be necessary, and the resulting changes to its final form after implementation, depend both upon the context in which it is implemented and the unique individuals engaged in the implementation process.

### Undergraduate students in the implementation of blended learning

Prior research on blended instruction strongly supports the efficacy of blended learning in higher education (Halverson et al., 2014; Means et al., 2009; Porter et al., 2014). Yet, there are few examples of literature discussing the evaluation of blended learning in context with implementation strategies that are specific to such innovations (e.g., Brown, 2016; Taylor et al., 2018a). In addition, those studies specifically targeting the implementation of blended learning rarely examine blended learning environments as a whole (Porter et al., 2016). Instead, they rely heavily on literature related to the dissemination, implementation, or use of specific educational technologies by new faculty or at new institutions.

As a result, formalized frameworks and models of blended learning implementation tend to feature students as a strictly environmental factor (e.g., Brown, 2016) when they feature students at all (e.g., Porter et al., 2014). This is somewhat surprising, even if it follows the general trends of implementation literature. Students’ self-regulated or self-directed learning behaviors are widely recognized as a critical component driving student success in blended learning environments (Kintu et al., 2017; Stacey & Gerbic, 2008). This implies that student interaction with blended learning innovations not only influences the implementation process, but directly contributes to the implementation’s success.

### Student resource adoption and self-direction in blended learning

Though research that formally examines student agency in context with processes of educational change remains rare, many studies have examined what factors might act as motivators or barriers to student engagement in the context of newly introduced, research-based, educational innovations. The expectancy-value model, for example, frames decision-making in terms of students’ perceptions regarding resource *availability*, *applicability*, and *quality*: students weigh these factors alongside their learning needs to determine the potential value of each resource they encounter (Makara & Karabenick, 2013). The Technology-Acceptance Model has likewise been widely applied in education research and frames students’ technology adoption decisions in similar terms; predicting students’ attitudes and engagement behaviors based on the resources’ *perceived ease-of-use* and *perceived usefulness* (Davis, 1993). Speaking generally, we can expect students’ adoption decisions to heavily depend on their perceptions regarding how *valuable* a resource is (i.e.: how much relevant help it can provide), and perceptions regarding how *easy* a resource is to engage with. For example, students with a high degree of digital literacy might view digital learning resources as being particularly easy to use (Sayaf et al., 2022). Likewise, students whose peers speak highly of certain resources might see more value in utilizing those resources themselves due to social influence (VanDerSchaaf et al., 2021). However, these studies tend to revolve around assessments of student adoption and engagement decisions. To examine how students go on to further implement the resources they choose to adopt (i.e.: studying how those adopted resources are adapted and utilized in the long-term), we must look beyond literature on adoption and instead examine *learning behaviors*.

Self-directed learning describes the process through which students select, plan, carry-out, and evaluate the efficacy of their own learning experiences. In self-directed learning, choices regarding *when* and *how* to engage with specific opportunities for learning lie partially with the students, rather than being wholly directed by their instructors. Therefore, self-directed learning relies heavily on students’ own internal motivations to learn, understandings of how learning works, and abilities to plan ahead (Litzinger et al., 2005). These students must be able to set their own goals, engage actively and metacognitively in the learning process, and evaluate their own learning outcomes (Jossberger et al., 2010).

Self-directed learning readiness has long been discussed as an essential contributor to student success and positive student perceptions of learning in blended environments (Ausburn, 2004), and engaging in blended learning can help students to develop their own self-directed learning aptitude (De George-Walker & Keeffe, 2010). However, blended learning does not *automatically* teach students how to effectively regulate their own learning (Adinda & Mohib, 2020; Sirakaya & Özdemir, 2018). Instructors must intentionally design for the enhancement of students’ self-directed and self-regulatory learning behaviors for development to reliably occur (Adinda & Mohib, 2020; Van Laer & Elen, 2020). Van Laer and Elen (2020) published a list of curricular design attributes that typify the facilitation of self-direction in blended learning. Together, these attributes are intended to foster students’ internal motivation, facilitate the adoption and use of blended learning resources, and encourage metacognitive and reflective learning practice in the presence of blended learning innovations (Van Laer & Elen, 2020). Put another way, instructors in blended learning are encouraged to empower their students, enabling them to better adopt, adapt to, and utilize their wide range of resources. Thus, implementing blended learning in a way that targets student self-direction involves adopting methods that treat the students *as implementors themselves*.

## Theoretical framework

Implementors, innovations, and their environments interact in complex ways during the implementation process. In this study, we employ Actor-Network Theory (ANT) as a theoretical framework to help better confront this complexity. In ANT all stakeholders, participants, technologies, and environments are conceived as being *actors* connected through mutual interactions (Latour, 1984). In the same way that people can act on objects around them to define the object’s purpose, uses, or roles, objects are theorized to act upon people to inform their own use (Tatnall & Davey, 2015). This can make ANT especially useful for the examination of innovation and implementation efforts (Fenwick, 2011; Harty, 2010). ANT provides a means of conceptualizing interactions between the various components and stakeholders involved in the implementation process. Within the scope of ANT, implementation becomes a process of renegotiation of roles and connections involving innovations, users, and their surrounding environments (Latour, 2005).

By adopting ANT, we are intentionally considering students as controlling actors, with each student working to influence their immediate learning network (Aheto, 2017). When given the agency to implement new, innovative approaches to learning, students attempt to change their learning networks in ways that incorporate those innovations in roles that are comprehensible and useful to the students themselves. In ANT, implementation is represented by the process of translation. Translation is the alteration of existing networks as certain actors attempt to exert control over their surroundings, altering them and attempting to redefine the roles of other actors as desired (Latour, 2005). However, these controlling actors (or implementors) can also be guided and changed throughout their work due to the influence of other actors, including the innovation they wish to implement.

In this way, the process of implementation is framed as a renegotiation of roles, one which may require compromise from any or all of the actors involved. Such processes of negotiation are well documented in implementation literature even outside the context of ANT. The concept of *mutual adaptation*, for example, has existed in discussions of business management for decades (Leonard-Barton, 1988). Incorporating ANT as a theoretical framework can help researchers identify and interpret mutual adaptations by allowing all the actors involved in the network, whether human, technological, or otherwise, to demonstrate agency during the implementation process. For example, ANT has proven useful in the study of Information and Communications Technology, disrupting researchers’ prior understandings to identify complex interactions in the use of digital learning technologies (Arif et al., 2017) and online learning spaces (Rowan & Bigum, 2003; Tatnall, 2019).

We are not employing ANT as a methodological approach. Rather, we are creating a hybrid study, incorporating ANT as a theoretical framework to inform our analysis and subsequent interpretation of findings in light of its sociomaterial perspective. As Fenwick and Edwards note in their introduction to *Revisiting Actor-Network Theory in Education*, “this practice of ANT hybrid is becoming more the norm than the exception” (Fenwick & Edwards, 2019: p. 3), and several chapters in their book follow a similar, hybridized approach. They reiterate from their previous book: “ANT offers, ‘a way of intervening in or interrupting education rather than simply a way of representing education’ (Fenwick & Edwards, 2019: p 4)”. Although ANT has been applied to Engineering Education Research (EER) before (Johri & Olds, 2011; Paledi, 2019), studies that intentionally treat students as controlling actors and implementors appear far more common outside of EER (e.g., Buhl, 2017; Luke, 2020; Pillai, 2017). By employing ANT, we intend to interrupt our current conceptions of implementation in education, opening the door to a wider range of possible interpretations.

## Methods

To study the experiences of individual students as they implement the *Freeform* environment, we employed a cross-institutional thematic analysis. We conducted this analysis in two parts. First, we analyzed the experiences of students at each separate institution, looking for commonalities and trends in the data from different implementations. Next, we took those initial codes and findings to compare them across the institutional populations, once again looking for clear themes and further interpreting them through the lens of ANT.

### Participant selection and institutional context

In approaching this multi-institutional study of implementation, we treated the student populations at each location as separate actor-networks. We assumed that all the students in these four distinct institutional populations considered their use of the *Freeform* environment to be of “relevance and personal significance” (Pietkiewicz & Smith, 2014: p. 10) to their experience in the course. Although students would experience implementation in individually distinct ways, every student could, in some way, speak to our phenomenon of interest due to their participation in the course. All students in each *Freeform* course were invited to participate in interviews, maximizing our number of potential participants given the relatively small population sizes. A summary of each participating institution and their corresponding sample sizes may be found in Table 2. By subsequently comparing our findings from participants in each context, we drew insight on any unifying, diversifying, or singular aspects of the experienced phenomenon of interest (the implementation of *Freeform*).

**Table 2:** Summary of participating institutions

Institution *n* = *# of student participants*	Abbreviation	Description (based on work by: Carnegie Foundation for the Advancement of Teaching, 2018)	Academic period
Purdue University—West Lafayette *n* = *12* *of 112 students*	PUWL	Public university offering 4-year baccalaureate, master’s and doctoral degrees. 42,000 + students, lower number of transfer students, primarily residential	Fall
Purdue University—Northwest *n* = *4* *of 46 students*	PUNW	Public university offering 4-year baccalaureate and master’s degrees. 12,000 + students, higher number of transfer students, primarily nonresidential	Summer
Trine University *n* = *14* *of 58 students*	Trine	Private university offering 4-year baccalaureate and master’s degrees. 4,000 + students, lower number of transfer students, highly residential	Spring
McGill University *n* = *9* *of 55 students*	McGill	Public university offering 4-year baccalaureate, master’s, and doctoral degrees. 39,000 + students, lower number of transfer students, primarily residential	Fall

All implementations of *Freeform* included in this study were within the context of engineering dynamics courses, and all occurred prior to the arrival of COVID-19 (years have been deidentified to preserve anonymity). All institutions received their own instantiations of the *Freeform* course blog (a course website containing digital learning resources), although some instructors, like in the case of Trine, chose to distribute *Freeform* resources using their own LMS. Decisions regarding the use of supplementary instruction or other out-of-class teaching activities were left to the discretion of each instructor.

### Data collection

Data were collected using a semi-structured interview protocol during in-person visits to each institution. The interview questions were written to address students’ experiences with learning resources and pedagogical approaches characteristic of *Freeform*, highlighting relevant stories and personally significant moments of experience. In addition, an initial set of questions asked students to describe themselves and their home institution, helping to establish the context for their experience*.* Interviews lasted 30–50 min, with duration largely driven by the interviewee. Interviews were held approximately two-thirds of the way through the semester, and participation was incentivized by individual $20 gift cards. After recording, interviews were sent to a third-party service for transcription.

A portion of our data (from PUWL) has been used in previous research (Evenhouse et al., 2020). By employing a new theoretical framework and broadening the scope to our multi-institutional dataset, we expect to discover new insights from these students’ stories and draw fresh comparisons across contexts.

### Data analysis

The structure of our analytical process drew heavily from Braun and Clarke (2006), following their step-by-step guide to thematic analysis while taking advantage of their framework’s flexibility. Analysis began with immersing ourselves in the dataset, reading through each transcript in its entirety and comparing their accuracy against the interview recordings. Inspired by Kirn and Benson (2018), we concluded this initial read-through by creating summary descriptions of each of our participants. This helped us to keep the individuality and personality of each of our participants in mind during subsequent analysis. Next, our first round of coding followed an in vivo approach, taking direct quotations from the students’ interviews and using them as initial codes (Saldaña, 2013). These codes were then iteratively grouped, described, and categorized into sets of emergent and clustered themes through repeated engagement with the dataset, as well as with our participant summaries and other notes (Braun & Clarke, 2006; Pietkiewicz & Smith, 2014). This process was continuously accompanied by analytical memo writing to inform creation and interpretation of results (Creswell & Miller, 2000).

In contrast to the inductive nature of our initial analysis, our subsequent comparison across institutional contexts was, to use the terminology of Braun and Clarke (2006), far more theoretical. Rather than allowing themes to again inductively emerge from the data, our team approached previous codes and themes through the lens of ANT, first identifying key actors in the students’ learning networks and subsequently examining their roles in the students’ experiences of implementation. The resulting high-level themes categorize and describe the actors themselves using key points of similarity or difference exhibited across institutions to clarify each actors’ influence on the implementation experience.

### Key limitations

Often, researchers employing thematic analysis continue incorporating data until initial codes and emergent themes reach *saturation*, a state wherein the inclusion of additional data fails to produce new insights. However, the number of participants required to reach saturation has proven extremely difficult to determine, with typical recommendations for small populations hovering around 10 participants (Fugard & Potts, 2015). While the total number of students included in this study (39) exceeds typical guidelines, participants are spread across four different instantiations of the *Freeform* environment. This makes it difficult to demonstrate saturation at any one institution, especially in the case of PUNW (*n* = 4). While our findings demonstrate clear differences in student experience between contexts, allowing for practical comparisons to be made, these findings should not be assumed to be freely generalizable. Rather, we hope that this study encourages readers to reflect upon their own experiences of implementation in new ways, guided by the subsequent analyses and informed by our results.

We approached analysis from an intentionally constructivist perspective, acknowledging the experiences of each student as individual, storied, and deeply complex (Ekebergh, 2009; Noon, 2018). As such, we took care at each stage to ensure that themes contained honest and accurate representations of our participant’s stories. Therefore, the themes below do not elucidate one common, universally defining aspect of implementation experience. Rather, we are using these themes to describe trends in the stories we collected, concisely representing a subset of illustrative, unique experiences and interpreting through the lens of ANT. We remind readers of this individuality of experience as they engage with our results and connect this work to their own contexts and experiences.

### Positionality statement

In addition to the limitations inherent to our research, we encourage readers to keep our own positionality as authors in mind when engaging with this work (Secules et al., 2021). One of our co-authors (JFR) was an original developer of the *Freeform* environment, and our team has been conducting research within the context of *Freeform* for more than 5 years. As such, we have developed understandings and expectations regarding its effects on student experience. We have no doubt that, despite our best efforts to bracket our assumptions (Fischer, 2009), our research and later findings will be colored by these previous research efforts. We recommend that readers contextualize this study within prior findings as they interpret and apply our results to their own contexts.

Our current interest in students’ experience and agency has taken years to develop. Research regarding *Freeform* has slowly shifted from early efforts centered on program development and evaluation to a more nuanced, intentional, and empathetic engagement with student experience. As tenure-track faculty (all PUWL faculty members were tenured at the time of writing), researchers, and former undergraduate students ourselves, we are each invested in the effort to better understand and improve the learning experiences of engineering undergraduates. However, due to our positions as paid researchers at a research-intensive university, we necessarily inhabit a position of privilege and perceived authority in our interactions with student (and perhaps faculty) participants. In addition, most of our team identifies as white or as men, labels which bear their own inherent privilege. The ramifications of this privilege and perceived power naturally extend to the collection of data and the creation of our research products, as the findings depicted here are interpreted and represented based on our own academic insight. The data represented here have been filtered multiple times, and in multiple ways, through our own scholarly lens—a fact which should be considered when engaging with this study. Though we have tried to empathize with the experiences described here as best we can, and to depict stories in an authentic manner, our analysis cannot relate the unmediated voices of our student participants.

## Findings

The results of this study are presented in two parts. First, we describe our findings at each institution, representing student experiences in the context of distinct implementations of blended learning. Second, we compare student experiences and themes across institutions, looking not only at students’ reported experiences, but also interpreting how they acted and interacted within their context and with the actors around them. Through this, we establish a cross-institutional perspective on the implementation of blended learning in the formation of individual students’ learning networks.

### McGill University (McGill)

Student interviews were collected during *Freeform’s* first semester at McGill University. According to student participants, dynamics was notorious within the McGill student community for the course’s difficulty. Many students reported that the course had high attrition, even saying that dynamics acted as a weed-out course for the ME program. While dynamics is widely acknowledged in literature to be challenging (Goldfinch et al., 2008; Martín-Blas et al., 2010), the number of interviewees who took time to call out their course’s difficulty was surprising. Most interviewees contextualized their experience in the *Freeform* environment by juxtaposing their personal, positive stories against this preexisting narrative of failure. In our first theme from McGill (as shown in Table 3), many students tied current successes in the course to the new structure brought to their dynamics curriculum. The blended approach and its intentionally aligned resources were often credited with students’ perceived improvements over their prior expectations.

**Table 3:** McGill institutional analysis—Theme 1

McGill Theme 1 *and sub-themes*	Representative quotations
Uprooting an institutional narrative s = 8	“I've heard horror stories about how it was before. It was a total mess of a course, I heard, just so much studying for so little return. The averages were very low before.” (McGill: Student 8)
*Narrative of failure and institutional pressures* *s* = *7*	“I expected the course to be very difficult. A lot of people at McGill, there's a rumor going around that about 30% of students fail dynamics usually because it's such a hard course…” (McGill: Student 4)
*Feelings of surprise and appreciation* *s* = *6*	“Yeah, this semester I really wanted to participate in this study because I love it. I love the things, like the [lecturebook], and I feel like it's awesome, honestly.” (McGill: Student 7)
*Attributed to Freeform structure, content alignment and progression* *s* = *5*	“…actually it's been going really, really great. I love how it's structured, in terms of … Just, like, we have our book that I bring to every class and I really base everything on it.” (McGill: Student 9)

Students often highlighted their individually accessible course resources and their instructor as the most important contributors to their counter-narrative experiences of success, leading to a second theme (see Table 4) that builds upon the content of the first. Each of the students’ sources of help took on a distinct role: course resources provided students with a unique opportunity for personal growth, and their instructor facilitated the use of those resources by incorporating them into students’ in-class experiences via active and collaborative pedagogies.

**Table 4:** McGill institutional analysis—Theme 2

McGill Theme 2 *and sub-themes*	Representative quotations
Resources as an opportunity for growth s = 8	“I think it's changed the way that I use online resources, and also the way that I interact with my peers… it forces me to go interact with my peers to actually see whether they have similar answers or different answers.” (McGill: Student 4)
*Resources allowed students to get ahead and self-direct* *s* = *8*	“I know what I can do. There is material so that I'm never like, "I just don't know how to study." I know how to study, I have material available, I have more material than the other year, I know what I can do.” (McGill: Student 7)
*The variety of resources helped students grow as learners* *s* = *6*	“…I would try and find past exams and see what the prof liked to ask, and try to deduce what he's going to ask in the future. Whereas, this [time] I think I'm actually learning the subject material more.” (McGill: Student 8)
*Students actively sought out or pursued additional resources* *s* = *5*	“… I'll tend to just Google it and the first ones that come up will be large physics forums where someone's like, "Hey, I don't understand why this works," and you'll get 20 or 30 responses. You get a large variety of it. And then you can pinpoint the differences in everyone's, so it gives you a large amount of different explanations…” (McGill: Student 1)

The majority of student participants at McGill were heavily invested in their use of out-of-class learning resources, seeing these resources as an opportunity to improve and advance their learning. Few had previously experienced similar blended learning environments, but this did not prevent them from trying to take full advantage of their resources by seeking out new opportunities for engagement. Many students reported that they used a wide variety of resources to find help and aid learning outside of class, and that the experience had taught them new lessons about how to learn and study effectively. Some went so far as to seek out additional non-*Freeform* resources by using dynamics textbooks from prior years, public online discussion forums, past exams, and student discussions on the PUWL course blog.

Interestingly, few students brought up their instructor as an individually accessible learning resource. The course instructor was more often portrayed as a facilitator of resource engagement rather than as a resource themselves. Many students highlighted their instructor’s efforts to encourage collaboration and engagement with educational technology, but few reported or recommended that interactions with their instructor outside of class were particularly helpful, or even accessible, leading to our final theme from McGill University (see Table 5). However, those students who *did* report using their instructor as a learning resource were eager to share their experience. Two students reported using their instructor as a resource on a regular (at the least, weekly) basis, making them a somewhat vocal minority within our sample. Similarly vocal minorities were not present in the other themes from our McGill dataset.

**Table 5:** McGill institutional analysis—Theme 3

McGill Theme 3 *and sub-themes*	Representative quotations
Instructor as a facilitator (rather than a resource) s = 8	“At the beginning of the year, the professor mentioned that he's very supportive of communication in the class. He wants students to talk to each other, and if they haven't understood the concepts properly then he suggests that students talk between them.” (McGill: Student 3)
*Brought blended and innovative methods into the classroom* *s* = *8*	“So usually the first half there's an interactive response thing, going through problems related to what we did the previous class. So usually he has us try them in groups for a little bit, and then we'll do it together.” (McGill: Student 2)
*Promoted collaboration and overcame barriers to engagement* *s* = *7*	“It opens more of a discussion… You want to call it a community, it can be a community of islands. Everyone has their own smaller community. Then, every once in a while, everyone will join at once.” (McGill: Student 1)
*Instructor was not often a resource outside of class* *s* = *5*	“I would say, because a lot of professors, especially at McGill, seem to be quite research-oriented. So they don't really care about teaching, right? They just are here to do their own research.” (McGill: Student 8)

### Purdue University—Northwest (PUNW)

The impact of extracurricular influences on the experience of students at PUNW was by far the strongest out of any of the institutions studied, laying out a compelling narrative for our first theme despite low student participation (see Table 6). During an early visit, an informal poll during class indicated that more than 90% of the students in the course were working 40-h weeks on top of their academic obligations. This included jobs inside and outside of engineering: some were taken as internships, while others were simply income in unrelated areas used to pay for tuition. Our study at PUNW not only occurred during the first instantiation of *Freeform* at the institution, but also during a Summer semester. As such, this extracurricular influence may have been, in part, due to the timing of data collection. However, from our interviews it appears that students at PUNW regularly expected to have home and work obligations outside of their academic load. Our only participant who was not working a full-time job had in fact lost their job several months prior and had yet to find a new position.

**Table 6:** PUNW institutional analysis—Theme 1

PUNW Theme 1 *and sub-themes*	Representative quotations
Prioritization of efficiency in the face of extracurricular pressures s = 4	“[In a] typical work week I work 40–45 h, but as of recently, I've been working 50 h, around there, a week. A lot of it tends to be extra hours on the weekend, so that kills my time to do homework. Besides working, I'm doing two other summer courses, and I'm taking care of my mom who's essentially disabled… It's kind of been tough with that, just to manage time and do different things that I normally wouldn't be doing.” (PUNW: Student 3)
*Unfortunate nature of student’s forced expediency* *s* = *4*	“Well, a lot of people have internships and everything else so, I'm used to just working with a group of friends and stuff. But since a lot of peoples’ schedules aren't flexible, this was like, a situation where we had to do homework by ourselves.” (PUNW: Student 4)
*Identification of inefficient resources* *s* = *4*	“The [discussion forum], I don't really see a use for that. Most people don't post on it. It's essentially like the discussion forums [on] Blackboard, and those rarely are used, here at least. I think the [discussion forum] portion [of the blog] should kind of be eliminated for the most part unless the professor was answering more on there.” (PUNW: Student 2)
*Identification of inefficient instruction* *s* = *4*	“Doing examples in class is fine, but when everyone is just watching the video on their phone, it's not really doing anything, everyone's just sitting around talking. That's time that could be used otherwise.” (PUNW: Student 2)
*Resources allowed for self-directed learning and engagement when needed* *s* = *3*	“…[the professor] will do two or three problems from the end of the chapter and then he'll make us do one. Then that's like, oh crap. He was showing us how to ride the bike. Now we have to ride the bike. We have to learn the steps and how to do them ourselves.” (PUNW: Student 1)

Students at PUNW were quick to notice when they encountered difficulties or barriers to learning that they deemed to be unnecessary or avoidable. Accompanying their personal needs for expediency when doing academic work, students were quick to point out teaching methods or even learning resources which they considered to be counterproductive, difficult to understand, or misaligned with the course or its assessments. Collaborative learning opportunities were often discussed in this context. In-class collaborative learning often required the instructor to move between multiple groups of students to provide them with targeted advice and feedback, a process which limited the number of students they could provide instruction to at any given time. At home, students were often forced to do homework at odd hours, or on a limited timetable, making it impossible for them to wait for responses to their discussion forum posts, emails, or even text messages.

However, every interviewee also highlighted how the broad range of resources provided by the *Freeform* environment helped them expediently complete homework assignments and allowed them to address questions as needed outside of class. Due to their work and school schedules, many students were forced to do homework late into the night. The solution videos and other easily accessible learning resources allowed students to find the help they needed, regardless of the time of day.

The videos helped in other ways, as three of the four interviewees discussed how the lecture example videos changed the ways they learned while in-class as well. Two discussed how the example videos could be used to help students overcome difficulties during collaborative in-class activities. Sometimes as the instructor moved between groups, multiple groups of students could require help simultaneously. Using the lecture example videos to provide guidance allowed collaborative efforts to move forward without requiring the instructor to personally address every question. Another student mentioned how the videos could be used to effectively make up for gaps in students’ concentration during lecture or to help students catch up to their peers after missing a day of class. Having an out-of-class instructional resource freed this student to pay attention more effectively during lecture and to make up for any incidental absences due to factors outside their control. Collectively, this interplay between in-class and out-of-class learning experiences gave rise to our second theme from PUNW, as shown in Table 7.

**Table 7:** PUNW institutional analysis—Theme 2

PUNW Theme 2 *and sub-themes*	Representative quotations
Out-of-class resources changed in-class engagement s = 4	“…this is entirely different from what I'm used to. It's always go to the lecture, you learn the lecture, you go online and do the online homework and you submit it. There's no intertwining that at all, but in this [course] there's a lecture; that's not all of it. You can go online, watch videos. You can look at examples, interact with people on the blog. It's so much deeper than a regular class setting.” (PUNW: Student 1)
*Effective supplement to learning that occurred in-class* *s* = *4*	“I enjoy it. You know, it's really focused on like the concepts and the theories and everything. It goes into it rather than, you know, we're just given the homework. 'Cause you know, we're given homework, but then it isn't really explained. Whereas you know here, we have a lot more resources to use, and it's helpful.” (PUNW: Student 4)
*Facilitated learning both inside and outside of class* *s* = *3*	“Previously in last semester… he just [wrote notes] on the board, and you take notes that way. With this course, I've noticed for myself I learn better if I just watched and I listened and I don't have to write down frantically all of his notes. It's awesome that all of the notes are right here [online and in the lecturebook] so I can look at him and what he's doing and understand.” (PUNW: Student 1)

### Trine University (Trine)

Our student participants at Trine talked extensively about the relationships they had with their institution and its members. In most cases, students took time to highlight the supportive and community-oriented culture cultivated at Trine. This broader sense of community was one of the biggest factors that initially drew our participants to the university and was frequently cited as a strong contributor to their overall academic success, giving rise to our first theme from Trine University (see Table 8). This was even true for participants who did not identify as particularly collaborative learners. In fact, several students who discussed the Trine community as an important aspect of their current learning experience preferred to work alone when outside of class. Even these students discussed the importance of the close interactions and personal connections, with instructors and peers alike, that had been fostered by the small campus environment at Trine. All students, to some extent, discussed the intentional culture of community support that their institution embodied.

**Table 8:** Trine institutional analysis—Theme 1

Trine Theme 1 *and sub-themes*	Representative quotations
Community investment in individual success s = 14	“What sets Trine apart is the individual courses in a way. Your courses are smaller so you can get more individualized help and attention if you need it. The professors are always willing to have a chat with you or help you with any problems you're having… It's a very individualized experience. I really enjoy that. They're very focused on your success.” (Trine: Student 3)
*Group oriented and collaborative institutional mindset* *s* = *12*	“Some days I'll work with students I don't have any classes with. I don't have any classes with them, but I know them from other sports or other previous classes. I work with people I don't really know their names. It's just a friendly environment.” (Trine: Student 14)
*Frequent interaction facilitated by shared spaces* *s* = *10*	“Maybe just the fact that since it's a smaller school I'm going to walk in the café and I'm definitely going to know someone there, so I'm not going to ever have to eat by myself. I have multiple classes with people that I know, so we can go back to the same place that we live and work on the same homework and whatnot.” (Trine: Student 1)
*Shared accountability amongst the community* *s* = *9*	“Yeah. Anytime there's homework due, we all get together. We talk it out. We do all that stuff. Our professor’s actually really good about this. He comes in every Sunday and answers questions that we have. We're all in there together. We're all figuring it out. We're all doing stuff together.” (Trine: Student 9)

Although the semester included in this study was not the first time *Freeform* had been used to teach dynamics at Trine, neither of the instructors at Trine had used the blended environment previously. The blended resources, pedagogical approaches, and even notation were largely unfamiliar to the instructors themselves. In contrast, the community-focused culture at Trine meant that students were very familiar with their instructors and comfortable looking to their instructors for help both inside and outside the classroom. Students described faculty as “approachable”, “caring”, and “available”, conveying a general expectation that Trine faculty were ready and willing to engage with their students to help them to succeed. As a result, many students expressed negative emotions regarding outside influences that they felt inhibited their instructor’s ability to teach. In the case of our research, this included any perceived impositions originating from the *Freeform* environment or its developers from PUWL. Our second theme (see Table 9) combines these observations—highlighting both the value of the students’ instructors, and the frustration that grew from seeing those instructors constrained by an outside influence.

**Table 9:** Trine institutional analysis—Theme 2

Trine Theme 2 *and sub-themes*	Representative quotations
Value of the instructor as a learning resource s = 11	“All the classes are taught by professors; we don't have TAs or anything like that. There's always office hours… Our dynamics professor, specifically, he comes in some Sundays even, to give kids help on the homework and stuff like that… The professors are all super helpful.” (Trine: Student 12)
*Expectations of faculty’s availability and willingness to engage* *s* = *11*	“All of the professors are willing to put in a lot of extra time to help you understand the material if you're willing to ask. That's probably the biggest thing.” (Trine: Student 2)
*Interactions extend beyond the classroom* *s* = *9*	“I do go talk to him quite a bit to get help. I use both, [my professor] and [the other dynamics professor] because he has office hours Sunday afternoon, five to seven. It's a good time if I'm trying to get my homework done… I go talk to [my professor] a decent amount too. Sometimes each one, the way they explain things is a little different.” (Trine: Student 14)
*Negative perceptions of influences that restrict instructor actions* *s* = *7*	“I feel like it's not quite as student success oriented as most of Trine’s other classes. As I said, if you go and talk to any of your professors, they're very willing to help at any point in time. [Our professor] has done that but because so much of it is online and he’s doing everything that he can with the lecture material and the examples that he’s given, I feel like it's a little limited in that regard if that makes sense.” (Trine: Student 3)

However, this does not mean that students refrained from using their new resources. Rather, students appeared to have a clear set of priorities when it came to studying outside of class, and they adapted their use of *Freeform* resources to suit those needs, giving rise to our final theme from Trine University (see Table 10). Students who highly valued interactive sources of help, including from faculty, reported that they continued to prioritize those sources of help despite the changes in their learning environment. Some students reported attending office hours of faculty other than their instructor when seeking additional help. Others reported attending office hours as a group or seeking help during odd times of the day or night, continuing with the same study behaviors they employed previously. However, students who worked alone for a significant portion of their study time frequently mentioned how the online solution videos (both the lecture examples and homework solution videos) empowered them to make the most of their time while studying alone, even if their overall study strategies did not exhibit any drastic changes. Many students also reported that their out-of-class and in-class engagement with learning resources mutually influenced one another. Students’ in-class collaborative learning activities, for example, fostered out-of-class collaborative engagement between peers by forging new social connections. As a result, the suite of blended resources provided by the *Freeform* environment empowered and enhanced the learning behaviors of many students, but was not capable of addressing all of the students’ learning priorities—especially those tied directly to their academic community.

**Table 10:** Trine institutional analysis—Theme 3

Trine Theme 3 *and sub-themes*	Representative quotations
New environment reshaped students’ actions, not priorities s = 11	“I'm a pretty quiet person so I don't usually go to professors' office hours so, with the online, that helps me a lot because I don't have to go and talk to my professor. I can get help from, I'm assuming, a doctor or a professor that's doing them online, and I can do it in the comfort of my own room and at the same pace.” (Trine: Student 8)
*Videos empower and enhance out-of-class learning* *s* = *10*	“I haven't ever had this many examples, which is better, I think. I really like it, because they're available to us, but it's not something that's really an assignment that I have to stress… Doing those examples without having that pressure of, ‘Oh, I need to get a good score on this,’ is helpful because I'm focused on learning.” (Trine: Student 11)
*Out-of-class and in-class learning activities guide one another* *s* = *9*	“I use [the lecturebook] in coordination with the videos, the lecture examples… The examples, those are kind of hard to look at, especially if I don't really understand what's going on in class: then I'll have poor notes. That's where the videos are helpful.” (Trine: Student 9)
*Blended resources address many needs, but not all needs* *s* = *7*	“…If I have a homework problem and I'm having issues with it, I always like to go online and try and find help from someone else, or examples that are similar or almost exactly the same. Maybe different numbers or whatever. And then I can go through and see how it's done. With this class, it's a lot harder to do that, because it's strictly through PUWL and even if there are other dynamics courses, they don't really match up with the way that this one's taught.” (Trine: Student 6)

### Purdue University—West Lafayette (PUWL)

The majority of the students interviewed at PUWL identified their institution, and in no small part themselves, by the challenging nature of their degree program. Many talked extensively about the difficulty of their courses, the challenges posed to them by their professors, and the perceived value of hard work, giving shape to our first theme from PUWL (see Table 11). Student interviewees often communicated how they had to work to live up to the expectations of their program and the dedication it took to pursue success. Many students also communicated that the community around them including administration, faculty, and especially their peers, were very supportive and willing to help. Although our interviewees rarely claimed to know all the students in their courses and often professed to be primarily individual learners, most interviewees were quick to complement their instructors and offered help to peers when needed throughout the semester.

**Table 11:** PUWL institutional analysis—Theme 1

PUWL Theme 1 *and sub-themes*	Representative quotations
Motivating effects of an institutional narrative s = 11	“There's a lot of pride in going to [PUWL], since you have your large alumni base… They've done a lot of great things, so then you have an obligation to live up towards that… trying your best to complete something, completing something to the best of your ability, and then making sure that it's your [best]…” (PUWL: Student 4)
*Challenge as a motivation for learning* *s* = *10*	“…as I'm sure a lot of people have said, it's not a simple course and it's not an easy major by any means, so you have to be willing to put the work in and do all of the things that are required of you to make it through the program.” (PUWL: Student 2)
*Unified community: extensive support alongside low-stakes competition* *s* = *8*	“It's like you feel important from your teachers, and they want you to succeed, but you also feel like you can work with a bunch of other personalities, in a way. You get a lot of experience working with other people who aren't necessarily like you… it's challenging in that way, but it's rewarding.” (PUWL: Student 5)

Students at PUWL emphasized how useful they considered their *Freeform* resources to be. In fact, most students’ discussions of their resources were not limited to the context of completing homework assignments; students described how their resources helped them to better learn dynamics concepts and content, or further helped them to become better learners. When rising to the challenge proffered by their curriculum, some students were accustomed to using outside resources such as alternative textbooks, online videos, or even educational websites like Coursera or Khan Academy to reinforce their learning. The blended resources provided by the *Freeform* environment addressed these students’ needs for additional learning opportunities in a similar way, but used content that was intentionally aligned with their actual course material. Many students found that the multiple representations of dynamics content offered by their blended and collaborative resources helped them to better understand and retain their engineering knowledge, which sets the tone for our second theme from PUWL (see Table 12). Although some students reported that it took time to get acquainted with their new blended resources, this expenditure was widely determined to be worthwhile.

**Table 12:** PUWL institutional analysis—Theme 2

PUWL Theme 2 *and sub-themes*	Representative quotations
Variety in resources as a facilitator of learning s = 10	“I would say it gives me a lot more opportunity to check my full understanding, rather than just check to see if my answer is correct. Because sometimes you can get in the middle of a problem and not realize that you are making assumptions that you're making, or not realize why an equation is the way that it is. And so, it's helped me more fully understand things, between both the videos with [the professor] explaining it. And also the blog because people will bring up things that I didn't think about.” (PUWL: Student 7)
*Utility of having multiple sources of information* *s* = *10*	“…it's a lot better because it gives you a variety of ways to understand the material, versus going [online] and I might find something that's not totally correct or not totally the way they want us to do it, or [not] something you need to learn for class. So, having that focused idea in a bunch of different varieties of [resources] helps a lot.” (PUWL: Student 2)
*Prevents the need for students to “blend” the class on their own* *s* = *7*	“…if I don't understand something [in another class], I'll have to go online and find out on my own. You know, just looking up random YouTube videos, and then sometimes you find something really good, sometimes you don't… with these online resources, because they were published by the professors, we know that they're going to be good and useful and relevant to what we're doing in the class” (PUWL: Student 4)
*Learning how to learn takes time* *s* = *7*	“I've invested probably more time in this class than any class this semester, and it's really helped me perfect my studying skills that I've learned to develop in the past but now I understand more—what I need to do to prepare for something or how I learn, or the amount of effort I put into something, which can be applied to any other class or project I'm working on.” (PUWL: Student 8)

Many students reported that *Freeform* connected in- and out-of-class learning experiences together in new ways, leading to our third and final theme from PUWL (see Table 13). This was most prevalent in discussions of how resources could address gaps in understanding. If certain topics were not covered during lecture, or if errors or lapses in concentration inhibited in-class learning, students felt confident that they could address their learning needs outside of class. Even complex topics that would typically require asking questions to their professor or peers could be solved at odd hours of the night through use of the online discussion forums or lecture videos. Instructors took time early in the course to encourage students to use all of the resources available, and many students subsequently benefitted from intentional engagement with blended and collaborative sources of help. Although each student could make their own decisions regarding what resources to use and why, the fact that each resource was intentionally aligned to the dynamics curriculum ensured that many different approaches to studying could be productive.

**Table 13:** PUWL institutional analysis—Theme 3

PUWL Theme 3 *and sub-themes*	Representative quotations
Mutual influences between in-class and out-of-class behavior s = 9	“I think just because more of an emphasis was put on [the blended resources] during class, like it was mentioned more. That you know, ‘oh if you're having problems with your homework, you should go to the blog,’ or, ‘we didn't do these examples during class so you should go and look at the example videos.’ So, I think the resources were about the same, but they were just used more.” (PUWL: Student 7)
*Connections between lecturing, notetaking, and studying* *s* = *9*	“Having those lecture example videos was just … I mean it … that set the whole course, I mean if I didn't know how to do a homework problem, usually there was one problem, maybe two that I could look at and try to pull bits of information out of, even if we didn't cover it in lecture.” (PUWL: Student 10)
*Collaborations and interactions in-person and online* *s* = *8*	“'Cause sometimes you don't have time to ask questions due to having a class afterwards, and sometimes you can't go to office hours… So it's really nice to just be able to go back and confirm my little questions [on the discussion forum], and then I can ask the big questions that need more time on with the professor.” (PUWL: Student 1)
*Addressing misalignments and poor execution* *s* = *7*	“So, probably like three times a week I use the lecture videos. It's usually to get help on homework or… Sometimes in class, if [the professor] makes a mistake at the end, and then I don't get time to fix it, I'll write a note at the top of my paper to go back and watch the video, because it's the same exact thing that he was going over in class.” (PUWL: Student 8)

### Theoretical interpretations: cross-institutional analysis

In interpreting our findings across the universities in this dataset, we identified actors who influenced the development of students’ learning networks and explored the roles those actors played in implementation. Our interpretation of these findings resulted in an examination of four primary actors: the institutional context, the subject of the implementation (in this case, the *Freeform* environment), the instructors, and the students themselves. Each of these actors influenced students’ resource usage decisions, thus impacting the development of each students’ learning network and study behaviors.

#### The role of the (institutional) context

The institutional context influenced students’ priorities and affective perceptions of their engagement with *Freeform* resources. Students at PUNW, for example, were heavily influenced by extracurricular pressures and responsibilities. This led them to prioritize resources that helped address gaps in knowledge and expeditiously solve homework problems. Although some participants at each of the four institutions reported a similar need for expediency, the students at PUNW spoke extensively about time constraints, the need to do homework quickly, and the importance of being able to find help when needed.

In contrast, very few students at McGill brought up extracurricular pressures. Instead, the majority of students viewed their new resources as an additional opportunity for personal growth. Students avidly pursued resources to support their own academic success, motivated by existing narratives of failure and pressures to perform reported at their institution. The institutional context also de-emphasized the instructors’ availability as an out-of-class resource, all of which led students at McGill to be especially invested in individually accessible learning materials on the blog or elsewhere. McGill was the only partner institution where multiple students reported using Visualizing Mechanics videos (pre-recorded mechanics demonstrations), and a large number of McGill participants reported using the PUWL blog as a supplemental resource. Students highlighted how they were able to approach dynamics concepts from many different perspectives by utilizing multiple learning resources, which in turn enhanced their learning and helped them to perform better on assessments.

Students at PUNW reported that they appreciated the structure that the *Freeform* environment imposed, highlighting how it tied together their in-class and online learning opportunities in a way that complemented the needs of their extracurricular responsibilities. Students at Trine, on the other hand, often discussed the imposition of *Freeform’s* structure in a negative light. The culture at Trine promotes personal engagement and individual investment, and students at Trine were accustomed to personalized instruction and interaction with professors. Perhaps as a result, the structure of the *Freeform* environment felt overly restrictive. Students felt as though the *Freeform* environment was preventing their instructors from adapting the course and its content to the needs of the students and the values of Trine.

Students’ implementation experiences at Trine were also complicated due to the use of an institutional LMS that took precedence over the *Freeform* blog. Students reported that many (but not all) of the videos that would typically be available on the *Freeform* blog site were also posted to their course’s LMS page. Some students even reported that they were not aware of the blog’s existence, as they had used the LMS to address all of their online study needs.

This could be contrasted with PUWL and its integrated instantiation of *Freeform*, as the environment had originally been developed for use at that institution. The resources and, more specifically, online presence of the *Freeform* environment was better established at PUWL than at any other institution. In fact, when students at other, partnered institutions searched the internet using phrases such as “*Freeform*” or “*Freeform* dynamics” the PUWL blog site would be the first to appear, *not* the blog site created for their own institution. In light of the maturity of this institutional implementation and its resulting online presence, many students at Trine, McGill, and even PUNW reported finding the PUWL blog and using its discussion forum as a source of help. Thus, these students had access to an additional resource. One discussion forum came to be used, albeit in a limited capacity, at all four of the participating institutions.

#### The role of the *Freeform* environment

Integrating students’ responses from all four participant institutions, the distinct “structure” of the *Freeform* environment was almost universally praised. However, students used the word “structure” to refer to multiple concepts. Some used “structure” to refer to the alignment and synchronicity between the various resources in the *Freeform* environment, describing the ways in which the lecturebook, videos, and even in-class lectures can be used to supplement, enhance, and interact with one another. Other students used the word “structure” to refer to the dynamics curriculum itself, describing the conceptual progression between chapters over the semester’s duration. This aspect of *Freeform’s* structure was most obviously represented through the content of the lecturebook, which provided a clear breakdown of dynamics concepts and acted as the students’ textbook (and notebook) throughout the semester.

Both aspects of “structure” drove students’ engagement with *Freeform’s* resources, but the reasoning behind those engagement behaviors varied. Students who promoted the alignment between the various aspects of the learning environment spoke extensively about how they could use their resources to complement each other, supplementing their learning and addressing misconceptions as they arose. For example, many students used the lecture example videos to clarify questions that arose during lecture. Others reported reading the lecturebook as a means of better preparing for class, or using the online discussion forums to further clarify dynamics concepts or problem-solving processes.

In comparison, students who promoted the structure of the course in terms of its curriculum and content discussed how it taught them both what they needed to learn *and* how they could go about learning it. Dynamics concepts built on each other sequentially and, rather than directly influencing *which* resources students engaged with, this informed *how* those resources were used to address relevant topics or ideas. This helped students to better understand underlying dynamics concepts, perform well on assessments, and develop a robust understanding of core engineering content knowledge.

The single factor that seemed to best facilitate both of these positive aspects of “structure” was the sheer variety of resources provided by the *Freeform* environment. Students who valued having multiple representations of their course content benefitted from a wide range of physical and digital resources (for more on the role of multiple representations see Ainsworth, 1999). Students who valued having a clear progression of concepts benefitted from the ease with which they were able to find the one or two resources that best addressed their needs. In either case, variety (in resources) helped to foster students’ learning activity, allowing them greater flexibility and confidence in shaping their own, personalized learning networks.

#### The role of the instructor

Instructors played an important role in guiding, encouraging, and facilitating students’ use of learning resources. This included resources that were accessed both collaboratively and individually, whether inside or outside of the classroom. Every institution had students who discussed a mutual influence between their in-class learning activities and their out-of-class studying. Instructors contributed to this process by introducing their students to innovative resources, facilitating collaborative and social engagement, and establishing norms for students’ engagement behaviors.

Students’ positive stories of their instructor’s facilitative actions most often described well-organized and highly structured in-class learning activities. For example, the collaborative in-class experiences at PUWL and McGill were well-received, with students highlighting how well they had been prepared for the activities and how easily they were able to find help from their instructor when needed. This can be contrasted with the students’ descriptions from PUNW, where some students felt ill-prepared or unsupported during their collaborative in-class activities. Unstructured deployment of innovations was typically frustrating to students, while highly structured activities with ample preparation and support were better received. This was especially true for interactive and collaborative experiences, such as those encountered during in-class group work or online via the discussion forums. This follows the same trends seen in prior literature, which demonstrate how students in blended learning prefer learning resources that are well organized and for which they have been adequately prepared (Finelli & Borrego, 2020; Martin et al., 2020; Taylor et al., 2018a).

The importance of instructors’ planning and structure can also be seen through their impact on the perceived alignment among resources and learning opportunities encountered in the course. Alignment depended heavily on the actions of the instructor, e.g., how they utilized the lecturebook material, what variable notation they used when writing, and whether they incorporated digital resources like videos into the classroom. Unfortunately, it appears that instructors’ influence on perceived alignment was most obvious in its absence. Students were quick to notice when there were inconsistencies between the content presented in-class and the content they encountered using other resources in *Freeform*. Such inconsistencies forced students to make decisions regarding which sources they could trust, or which way was the “right way” to learn the course material, drastically complicating the engagement decisions they had to make while studying.

#### The role of the students

At each institution, we saw examples of students who chose not to implement resources that are typically considered to be essential parts of the *Freeform* learning environment. This is not necessarily surprising, as previous works found numerous distinct resource usage patterns and behaviors among their student participants. However, seeing vast differences in student engagement within populations at multiple institutions has clarified and emphasized the important role that students take in determining their own, personalized use of the innovations they encounter.

For example, most students at McGill reported overwhelmingly positive experiences with, and perceptions of, the *Freeform* lecturebook. Juxtaposed against an institutional narrative of failure, many students commented on the utility of the lecturebook and its accompanying lecture example videos. These comments could be simple and direct such as, “…just follow the lecturebook and you'll be fine” (McGill: Student 8), but some were more comprehensive.

“It's, like, I use it almost every day. I just read it over and over. To prepare myself for midterm assignments, I'm going to prepare myself with the dynamics [lecturebook]. For the final as well. I also use it all the time during class… Yeah, we call the dynamics book the Bible. It's really what we base everything [on]... Every time we have a question we look at the book, you know?” (McGill: Student 9)

Despite what would appear to be overwhelming support for the lecturebook, some students still chose not to use it. Upon reflection, these students could even acknowledge that they differed from the norm, saying for example, “Many people do write directly into the lecture book, but I personally write into a notebook of my own.” (McGill: Student 4)

Looking more broadly, there was at least one student from every institution who reported not using the *Freeform* lecturebook, which previous study has noted to be the most popular resource among students at PUWL (Stites et al., 2020). This illustrates that students at all institutions had agency to determine the people, practices, and resources that made up their own personal learning networks. It also highlights the importance of students’ ability to choose *not* to adopt, and thus not to implement, some of the resources they encounter. Students determined their own engagement behavior and study strategies in light of, or sometimes in spite of, any outside influence from their instructor, their institution, their peers, or otherwise.

In discussing their decisions and resulting behaviors, students often talked about their individual resources as filling a specific role: a role which either did or did not align with their own understanding of their individual needs and objectives. In addition, different students could perceive the same resources as filling different roles. For example, Student 13 from Trine chose not to purchase a lecturebook, justifying their choice by specifically referencing notetaking.

“Actually, I think this is a disadvantage for me. I'm a note taker. I learn by taking notes and, not regurgitating, but modifying what is put on the board and representing what it means in my personal perspective. In this class, it's actually been harder for me because it's filling out notes in that book, in the dynamics [PUWL] book, and it's less of me modifying and thinking.” (Trine: Student 13)

The student above determined that the lecturebook held little value for them due to how closely their notetaking had to align with their lecture content. In contrast, most of our interviewees valued the lecturebook for this same quality, highlighting how the lecturebook could interact with the pre-written lecture example videos to reinforce learning outside of class. Of course, students’ interactions with the lecturebook serve as only one example. Many students reported avoiding particular resources such as the online discussion forums, peer collaboration, or instructor office hours for reasons tied to their own learning goals and preferences.

However, far more often we encountered examples at each institution of students who went out of their way to engage with new resources or opportunities for learning—students who adopted, adapted, and implemented the innovations they encountered in the context of their own study behaviors. These students changed the resources they engaged with, or changed the *ways* in which they engaged with their resources, in an effort to improve their own learning. Many students reported “learning how to learn” as a result of their self-directed engagement with the *Freeform* environment by not only tailoring the resources of the environment to fit their needs, but also changing their approach to learning in order to better interact with new innovations. This metacognitive engagement is a hallmark of self-directed learning (Jossberger et al., 2010) and was demonstrated in students’ use *and* non-use of their blended learning resources.

## Discussion

### Practical observations

Students across every institution expressed appreciation for the “structure” of the *Freeform* environment. This is a theme that we had observed in previous work on the PUWL campus (Evenhouse et al., 2020), but seeing its emphasis across multiple institutions reflects broader research on the implementation of blended learning. Taylor and Newton (2013) collected data on an institution-wide implementation of blended learning, reporting that students appreciated “well-structured subjects and relevant and accessible learning resources” (p. 56), among a variety of other factors. This emphasized a need for implementors of blended learning to focus on “learning design and learning support, rather than technologies” (p. 57), and our findings reinforce this conclusion. Instructors at each institution in this study employed different technologies while teaching, and students employed a wide range of digital learning resources in their studies. However, students most appreciated those resources that aligned closely with the content of the course and their understanding of course assessments, highlighting the importance of students’ *perceptions of resource relevance* (i.e.: perceived value, or perceived usefulness) in both the design of resources and their implementation.

While this study has re-emphasized the importance of alignment and structure in blended learning environments, we are not suggesting that local adaptations and changes are detrimental to the implementation process. Rather, adaptations could help to reinforce alignment by correcting for contextual factors that the original developers could not account for. Our findings from Trine University testify to this: forcing instructors to adopt *Freeform* and its practices with an extremely high degree of fidelity would directly conflict with salient aspects of the institution’s prevailing culture. Students at Trine have an understanding that their instructors are both competent and caring, and that they will adjust instruction to their students’ benefit. Placing strict limitations on instructors’ ability to adapt would compromise the efficacy of the students’ most highly valued learning resources and could adversely affect their experience in the course.

### A new perspective on implementation

The ANT-informed findings in this study illustrate the value in viewing students as individual, controlling actors within the scope of their own learning networks. Based on our interpretation and a broader review of literature, students in this study go through individual processes of implementation by adopting and adapting the various innovations they encounter. This would suggest that the success of *institutional* implementations of blended learning depend, in part, on the implementation experiences and decision-making processes of *individual students*.

In prior study, we identified how the perceived availability of a resource, and the perceived relevancy of the help provided by that resource, directed students’ behavior in the *Freeform* environment, tying our participants’ engagement decisions in a resource-rich environment to contemporary models of student resource adoption (Evenhouse et al., 2020). Based on our findings here, the ways in which other actors influence students’ engagement behaviors seem to extend beyond the individual adoption decisions motivated by perceptions of availability and relevancy, or ease-of-use and usefulness. At the very least, this study hints at a complicated, contextual, and perhaps idiosyncratic web of factors which mediates the formation of students’ perceptions of, engagement with, and use of learning resources. Thus, this study supports using a nuanced, contextually informed approach to understanding the implementation experiences of undergraduate students in the presence of educational innovations. As students in this study described their approaches to learning both inside and outside of the classroom, they engaged with a variety of actors who impacted their decision-making processes and subsequent behaviors. In future study, researchers may benefit from not only examining the perceptions and self-directed behaviors of individual students, but also from exploring how these experiences are embedded within a larger network of concerned actors: human, institutional, or otherwise.

These conclusions call for an expansion of the ways we conceptualize processes of pedagogical change in undergraduate STEM education. Taking an instructor-centric approach has proven useful, and systemic reviews of literature have demonstrated both the nuance and complexity of implementation efforts of faculty (e.g., Liu et al., 2020). However, respecting students as independent actors in the implementation of innovations allows for a similarly nuanced discussion of students’ own engagement decisions and their subsequent implications for educational change efforts.

### A new role for instructors

ANT provides language to better discuss and situate these findings. In describing the implications of utilizing an ANT perspective, Latour writes, “…with ANT: the theory of action itself is different, since *we are now interested in mediators making other mediators do things*.” (Latour, 2005: p. 216–217, *emphasis ours*). In conceptualizing the role of instructors as mediators influencing mediators, and thereby acknowledging the agency of students in the implementation process, we gain new insight into what instructors can do to help students succeed in the innovative classroom. Rather than snapping students and learning resources together like puzzle pieces, instructors can change the way in which innovations interact with students and, in turn, direct the ways in which students interact with the innovations they encounter—an actor facilitating actors, and an implementor facilitating implementors.

The idea of faculty as facilitators of students’ in-class engagement and growth as learners is not a new concept. The facilitative efforts of faculty come up repeatedly in context with blended learning (Taylor et al., 2018a), collaborative engagement (Martin et al., 2020), and the use of online resources (Lewis & Abdul-Hamid, 2006). As such, we emphasize that students’ own processes of decision-making, adoption, and subsequent adaptation of new learning opportunities are especially important in light of the unique affordances and requirements of blended learning (Gedik, Kiraz, & Özden, 2012). In fact, Tang and Chaw (2016) concluded that students’ ability to adapt in the presence of technological innovations is a significant predictor of whether individual students are ready to engage with blended learning. Unfortunately, the role that faculty can play in shaping such adaptations remains relatively unexplored.

With students not only adopting new technologies, but engaging in entire processes of individual implementation, faculty could be said to own an additional responsibility: to act as facilitators of their students’ implementation processes and as catalysts for their metacognitive growth as learners. Though there are pedagogical resources and frameworks that can help instructors to foster such metacognitive growth (Gamby & Bauer, 2022; Van Laer & Elen, 2020), few have been intentionally extended to inform the broader implementation and use of other research-based educational innovations. ANT can help us understand how the actions of an instructor are not only connected to the statistical measures of engagement and performance of their students, but to the *actions* of their students as agents in the implementation process. Future work should interrogate this role of instructors as facilitators of student action, not only in terms of pedagogy, but in terms of student perceptions and behaviors in their personal interactions with educational innovations.

## Conclusion

Using thematic analysis, we examined students’ experiences of implementation in a blended learning environment applied to Mechanical Engineering dynamics courses across four universities. In contrast to a more typically instructor-centered perspective on implementation, we examined the role(s) of students as actors and agents of change who shape the adoption and subsequent expression of learning innovations. By intentionally examining students’ use of blended resources as a process of implementation through the lens of ANT, we described a number of actors who influenced our participants’ learning behaviors. The institutional context, the innovation being implemented, and the course instructors all contributed to the formation of students’ personal learning networks.

Students reported making informed and reflective decisions regarding which blended resources to use, sometimes rejecting popular resources due to their own personal learning needs. The other actors in the network helped to inform, shape, and facilitate students’ engagement behaviors. For example, many students were motivated to engage with new resources when there was an overarching structure, or obvious alignment, tying the content of their course, their assessments, and their blended resources together. Likewise, instructors could help to facilitate students’ engagement with new resources by utilizing or promoting them in class, demonstrating the structure or alignment of the course environment, or by maintaining clear channels of communication with their students for feedback, guidance, and support. Many students reported that the innovative resources they encountered helped them to “learn how to learn”, indicating that they not only adapted the resources they encountered to fit their needs, but also adapted their own habits and behaviors to better utilize resources that they perceived to be valuable and effective.

In conclusion, we believe there is value in further investigating the active role that students play in the implementation of innovations, even when those innovations are not explicitly participatory in nature. Simply examining student experiences with, and perceptions of, educational innovations may be casting students in too passive a role. Developing a more nuanced understanding of students’ behaviors and how they are influenced by other actors in their learning network could help instructors to not only successfully implement innovations, but also to better empower their students to succeed in the innovative classroom.

## Data Availability

The data sets generated and analyzed during this study are not publicly available due to the identifiable nature of the data.
